# Accurate emulation of steady-state and dynamic performances of PEM fuel cells using simplified models

**DOI:** 10.1038/s41598-023-46847-w

**Published:** 2023-11-09

**Authors:** Hossam Ashraf, Mahmoud M. Elkholy, Sameh O. Abdellatif, Attia A. El‑Fergany

**Affiliations:** 1 Electrical Engineering Department, Faculty of Engineering and FabLab in the Centre for Emerging Learning Technologies (CELT), The British University in Egypt (BUE), Cairo, Egypt; 2 Electrical Power and Machines Department, Faculty of Engineering, Zagazig University, Zagazig, 44519 Egypt

**Keywords:** Fuel cells, Electrical and electronic engineering

## Abstract

The current effort addresses a novel attempt to extract the seven ungiven parameters of PEMFCs stack. The sum of squared deviations (SSDs) among the measured and the relevant model-based calculated datasets is adopted to define the cost function. A Kepler Optimization Algorithm (KOA) is employed to decide the best values of these parameters within viable ranges. Initially, the KOA-based methodology is applied to assess the steady-state performance for four practical study cases under several operating conditions. The results of the KOA are appraised against four newly challenging algorithms and the other recently reported optimizers in the literature under fair comparisons, to prove its superiority. Particularly, the minimum values of the SSDs for Ballard Mark, BCS 0.5 kW, NedStack PS6, and Temasek 1 kW PEMFCs stacks are 0.810578 V^2^, 0.0116952 V^2^, 2.10847 V^2^, and 0.590467 V^2^, respectively. Furthermore, the performance measures are evaluated on various metrics. Lastly, a simplified trial to upgrade Amphlett’s model to include the PEMFCs’ electrical dynamic response is introduced. The KOA appears to be viable and may be extended in real-time conditions according to the presented scenarios (steady-state and transient conditions).

## Introduction

Currently, global concerns are directed towards investing in carbon-free energy sources due to the severe ecological impacts of fossil fuel-based energy sources^1,2^. Besides, the rise in prices of fossil fuel due to international conflicts and its limited existence have expedited the necessity to find sustainable and environmentally friendly energy sources^3^. Amongst such clean sources, fuel cells (FCs) are considered as a new booming application of renewable energy-based conversion technology^4^. Principally, FCs convert chemical energy into electrical and heat energies. Due to their higher conversion efficiencies, robustness, and almost zero emissions, FCs are compatible to be utilized in whatever application either in industrial, commercial, or residential sectors^5^. According to the electrolyte substance, FCs are divided into various types. Each one has unique features represented by the output power range, the operating temperatures, and the appropriate applications^6^. Particularly, proton exchange membrane FCs (PEMFCs)^7^, solid oxide FCs^8^, alkaline FCs^9^, phosphoric acid FCs^10^, and molten carbonate FCs^11^ are examples of FC’s types.

PEMFCs have gained wide popularity because of their attractive characteristics such as low operating pressures and temperatures, high power density, compact size, and no dynamic parts. However, the high cost of the catalyst hinders their market dominancy^4–6^. Moreover, the output voltage per cell varies from 0.9 to 1.23 V depending on the temperature value and the suppliant pressures, hence, a group of PEMFCs are connected serially to magnify the output voltage to a desire level. This group of PEMFCs is called a stack^12^. Additionally, their output voltage exhibits a non-linear relationship with the load current caused by the polarization losses. In other words, the PEMFC’s output voltage starts falling rapidly due to the activation voltage drop, then it decreases linearly because of the ohmic voltage drop, and finally it extremely decays due to concentration losses^13^.

Since modelling the PEMFCs suffers from a high degree of non-linearity, robust techniques are vital to precisely simulate the electrical characteristics of the PEMFCs, and hence, properly assess their performance. Thus, numerous researchers have proposed several models to describe the PEMFC’s operation from different perspectives^4–6^. Generally, PEMFC’s models are categorized as empirical, semi-empirical, and analytical models, as reported in^14–17^. In this article, a semi-empirical electrochemical model, proposed by Amphlett et al.^6,18^, is utilized to properly evaluate the simulated steady-state electrical characteristics of PEMFCs. During recent decades, Amphlett’s model has acquired a reputed acknowledgment as a result of its powerful ability to predict the electrical behavior of the PEMFCs in various operating environments^4–6,18^.

Nevertheless, the mathematical representation of such a model includes a set of undefined parameters that aren’t stated in the fabricators’ datasheets. These parameters need to be optimally estimated so that the model can perfectly emulate the actual behavior of the PEMFCs. Accordingly, vast attempts have been conducted to completely define the unspecified parameters of the PEMFC’s model. Basically, these attempts can be classified into conventional and artificial intelligence (AI)-based optimization ones^4–7^.

For instance, the techniques based on electrochemical impedance spectroscopy^19^, adaptive filter^20^, and current switching^21^ are representatives of the conventional trials. However, they aren’t broadly employed as their construction relies on the iterative techniques derived from different numerical approaches. Thence, the model’s startup conditions, complexity, and iteration steps are the dominant factors affecting the accuracy of such methods. On the contrary, the AI-based methods, represented by metaheuristic optimization algorithms (MOAs), are dependent on the specs of the computer processor on which the optimization task is carried out, which make them more reliable and effective. Consequently, a huge number of researchers have applied MOAs in estimating the unknown parameters of the PEMFC’s model^4–7,12,22^.

Specifically, artificial bee colony-differential evolution algorithm (ABCDEA)^23^, artificial ecosystem algorithm (AEA), ant lion algorithm (ALA), and multi-verse algorithm (MVA)^24^, and artificial rabbits algorithm (ARA)^25^ are examples of MOAs, involved in the PEMFC parameter estimation task. Besides, bi-subgroup algorithm (BSA)^26^, chaotic Harris hawks algorithm (CHHA)^27^, converged moth search algorithm (CMSA)^28^, circle search algorithm (CSA)^29^, evaporation rate water cycle algorithm (ERWCA)^21^, enhanced transient search algorithm (ETSA)^30^, and firefly algorithm (FFA)^31^ are applied for the afore purpose. Not only the previous-mentioned algorithms but also, gorilla troops algorithm (GTA)^32^, hunger games search algorithm (HGSA)^33^, improved chicken swarm optimizer (ICSO)^34^, improved fluid search algorithm (IFSA)^35^, Jellyfish search algorithm (JSA)^36^, lightning search algorithm (LSA)^37^, and many mor are employed for the same goal^4–6^.

According to the "no free lunch" (NFL) theory^38^, each optimization technique has advantages and disadvantages for particular jobs, hence there isn't a single algorithm that can solve all engineering optimization issues. Furthermore, there is still no conclusive answer, and it can be challenging to choose between optimization methods X and Y depending on factors like degree of non-linearity, non-convexity, multi-modality, separability of the control variables, high dimensionality, etc. The attempts will continue till such a response is received in these undertakings. Even though there have been many successful methods to establish these parameters, as indicated above, there is always potential for improvement to more precisely address the best PEMFCs stack model values.

It's self-explanatory from the previous short review that specifying the unidentified parameters of Amphlett’s model became a critical research area, at which the aforementioned algorithms struggle to attain minimum errors, lower computational burden, and superior statistical metrics. The earlier stated has encouraged the authors to assess the efficacy of a new physics-based MOA, named Kepler optimization algorithm (KOA) introduced in 2023^39^, in generating the ungiven parameters of four well-reputed PEMFCs’ stacks. It’s worth declaring that the KOA possesses significant features like a smooth transition from exploration to exploitation to escape from local minima trap, rapid convergence tendency, and lower execution time. It’s worth noting that based on the authors’ awareness, upon an accurate investigation, it’s the first employment of the KOA in the PEMFC’s parameter-specifying task.

It's time to call attention to the main contributions of this article, which include: (i) using and examining KOA’s performance to optimally assign the values of unknown parameters in the Amphlett's model, (ii) carefully examining four real-world study cases, Ballard Mark V, BCS 0.5 kW, NedStack 6 kW, and Temasek 1 kW, and (iii) Numerous statistical comparisons among the KOA optimizer and other recent and benchmark optimizers are performed, and (iv) Amphlett's model is modified to more accurately capture the dynamic behavior of PEMFCs stack before a real-world test case is evaluated for its dynamic responses.

This paper is organized as follows: Section "Introduction" introduces a summarized review and the motivation of the current endeavor. The mathematical formation of Amphlett’s model is deeply discussed in Section "Mathematical formulation of PEMFCs’ model". Section "Cost function allocation" announces the allocated cost function (CF) and its relevant boundaries. Section "Kepler optimization algorithm" illustrates the procedures of KOA. Various simulated test cases under different steady-state conditions, as well as executing some statistical tests to evaluate the KOA’s performance are revealed in Section "Numerical simulations and algorithm verification". A simplified trial to update Amphlett’s model so that it can describe the electrical dynamic response of the PEMFCs is introduced in Section "Dynamic assessment of PEMFCs stack". Lastly, Section "Conclusion and future prospective" announces the conclusion and the future perspectives.

## Mathematical formulation of PEMFCs’ model

As formerly-stated, Amphlett’s model is regarded as the most powerful method to simulate the dependency of the PEMFC’s terminal voltage on the load current through various steady-state cases^22–24^. In order to examine the PEMFCs stack in operation, Mann and Amphlett's PEMFCs model makes a number of simplifying assumptions^18,40^. Here are a few of these presumptions: (i) The fuel and oxidant gas compositions are constant and precisely blended throughout the entire cell, (ii) The electrodes completely consume the fuel and oxidant, preventing any buildup inside the cell, (iii) There is no gas mixing between the anode and cathode compartments because the fuel and oxidant gases run parallel to the electrodes, and (iv) The porosity and thickness of the material do not change over time, (v) The fuel and oxidant gases' temperature, pressure, and humidity remain constant across the entire cell, and (vi) the electrolyte is a perfect ion conductor with no limits on electronic conductivity or mass transfer.

**Figure 1:**
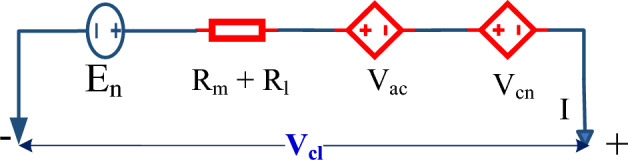
Equivalent circuit per cell.

In spite of the aforementioned, the Mann’s model can be extended to describe the transient behavior as well^24^. Amphlett supposes that the PEMFC’s terminal voltage is subjected to three voltage decays which are the activation, the ohmic, and the concentration voltage drops, as described in Fig. 1^29^. The next few statements concisely illustrate the model, as thoroughly described in the state-of-the-art. The terminal voltage per single cell ${V}_{cl}$ ($V$), is given by (1)^25^.

1 $${V}_{cl}={E}_{n}-{V}_{ac}-{V}_{oh}-{V}_{cn}$$

where, ${E}_{n}$ is the Nernst open-circuit voltage per PEMFC in ($V$), ${V}_{ac}$ is the activation over-potential in ($V$) as the startup chemical reactions are relatively slow, ${V}_{oh}$ is the ohmic voltage loss due to the total resistance of the membrane and external leads in ($V$), and ${V}_{cn}$ is the concentration over-potential due to the high water content in the membrane at heavy loading in ($V$).

As earlier-mentioned, to attain a high terminal voltage, $N$ PEMFCs are connected in series creating a PEMFCs’ stack whose terminal voltage is ${V}_{st}$ ($V$) and calculated by (2)^26^.

2 $${V}_{st}=N\cdot {V}_{cl}$$

Generally, (2) supposed that all the PEMFCs are identical and behaves the same^6^.

The ${E}_{n}$ can be calculated by (3) for $T\le 373.15 K$^27^.

3 $${E}_{n}=1.229-8.5\times {10}^{-4} \left(T-298.15\right)+4.3085\times {10}^{-5}\times T\left[ln\left(\sqrt{{({P}_{H})}^{2}{P}_{O}}\right)\right] $$

where, the partial pressures of hydrogen and oxygen are represented by ${P}_{H}$ and ${P}_{O}$ in ($atm$) and determined by (4) and (5), respectively^25^. $T$ is the PEMFCs’ stack operating temperature in ($K$).

4 $${P}_{H}=0.5\cdot {RH}_{a}\cdot {P}_{w}.\left[\left[\frac{1}{\frac{{RH}_{a}\cdot {P}_{w}}{{P}_{a}}\cdot exp\left(\frac{1.635I}{{A}_{m}\cdot {T}^{1.334}}\right)}\right]-1\right]$$

5 $${P}_{O}={RH}_{c}\cdot {P}_{w}\cdot \left[\left[\frac{1}{\frac{{RH}_{c}\cdot {P}_{w}}{{P}_{c}}\cdot exp\left(\frac{4.192I}{{A}_{m}{\cdot T}^{1.334}}\right)}\right]-1\right]$$

where, the load current and membrane useful area are denoted by $I$ and ${A}_{m}$ in ($\mathrm{A}$) and (${\mathrm{cm}}^{2}$), respectively.

${RH}_{a}$ and ${RH}_{c}$ express the relative humidities of the water steam at the anode and cathode regions, respectively. The anode and the cathode inlet pressures are indicated by ${P}_{a}$ and ${P}_{c}$ in ($\mathrm{atm}$), respectively. The saturated vapor pressure is symbolized by ${P}_{w}$ in ($\mathrm{atm}$), which can be given by (6)^29^.

6 $${log}_{10}({P}_{w})=0.0295\left(T-273.15\right)-9.18.{10}^{-5}{\left(T-273.15\right)}^{2}+0.0144\cdot {10}^{-5}{\left(T-273.15\right)}^{3}-2.18$$

Moreover, ${V}_{ac}$ can be computed by (7)^30^.

7 $${V}_{ac}=-[{\zeta }_{1}+{\zeta }_{2}\cdot T+{\zeta }_{3}\cdot T\cdot ln({C}_{O})+{\zeta }_{4}\cdot T\cdot ln(I)]$$

where, ${\zeta }_{z} ( z=1:4$) refer to semi-empirical factors in ($V,V{K}^{-1},V{K}^{-1},V{K}^{-1}$). ${C}_{O}$ symbolizes the oxygen concentration at the catalytic region in ($\mathrm{mol }{\mathrm{cm}}^{-3}$), which is formulated by (8)^31^.

8 $${C}_{O}=\frac{{P}_{O}}{5.08.{10}^{6}}\cdot exp(\frac{498}{T})$$

Furthermore, ${V}_{oh}$ can be expressed by (9)^32^.

9 $${V}_{oh}=I\cdot \left({R}_{m}+{R}_{l}\right); {R}_{m}={\rho }_{m}\left(\frac{l}{{A}_{m}}\right)$$

where, the resistances of the membrane and leads are denoted by ${R}_{m}$ and ${R}_{l}$ in ($\Omega )$, respectively. $l$ points out the membrane thickness in ($cm$). The membrane specific resistivity is represented by ${\rho }_{m}$ in ($\mathrm{\Omega cm}$) and determined by (10)^33^.

10 $${\rho }_{M}=\frac{181.6\left[1+0.03\left(\frac{I}{{A}_{m}}\right)+0.062{\left(\frac{T}{303}\right)}^{2}{\left(\frac{I}{{A}_{m}}\right)}^{2.5}\right]}{\left[\lambda -0.634-3\left(\frac{I}{{A}_{m}}\right)\right]\cdot exp\left(4.18\left(\frac{T-303}{T}\right)\right)}$$

It’s worth affirming that $\lambda$ refers to the water content in the membrane and its determination is a challenging issue because of its reliance on the cell-drawn current. However, a certain water content, at all possible operating conditions, is assumed unchangeable in this work.

Additionally, ${V}_{cn}$ is given by (11)^34^.

11 $${V}_{cn}=-\delta ln\left(1-\frac{I}{{A}_{m}\cdot {J}_{m}}\right)$$

where, $\delta$ is an empirical factor in ($V$) and ${J}_{mx}$ is the peak current density in ($\mathrm{A }{\mathrm{cm}}^{-2}$).

Lastly, the stack output power $\left({P}_{st}\right)$ can be determined by (12)^36,41^.

12 $${P}_{st}={V}_{st}\times I$$

At this moment, the reader can easily notice the seven unknown parameters are namely (${\zeta }_{1}, {\zeta }_{2},{\zeta }_{3}, {\zeta }_{4}, \lambda , {R}_{l} and \delta$) that are ungiven in the manufacturers’ datasheets^31–37,39^. These parameters are optimally generated using the KOA-based methodology to achieve an accurate simulation of the PEMFCs under various operating scenarios.

## Cost function allocation

Herein, the summation of square deviations (SSDs) between the actually measured voltages ${V}_{ms}$ and the model-based calculated ones ${V}_{cal}$ is adopted to optimally pick the values of the seven parameters. Since the SSDs, given by (13), is vastly employed in the literature^22–37,39^, and also to make a just comparison to the already-published algorithms, the CF is assigned to minify the SSDs, as depicted in (14).

13 $$SSDs=\sum_{m=1}^{n}{\left[{V}_{ms}(m)-{V}_{cal}(m)\right]}^{2}$$

14 $$CF=Min \left(SSDs\right)$$

where, n defines the number of the measured voltage-current dataset points.

Furthermore, the SSDs is susceptible to inequality bounds where each unknown parameter has its own lower and higher limits. It’s noteworthy to indicate that the KOA preserves these limits while searching for optimal values. Utilizing the SSDs values, the main goal is to significantly fit the recorded terminal voltages to the relevant computed ones by the KOA-based methodology.

## Kepler optimization algorithm

**Figure 2:**
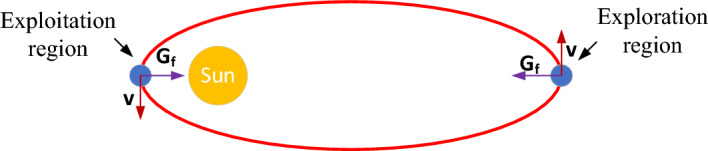
KOA’s exploration and exploitation regions.

Basically, KOA is a physics-based optimizer that imitates the planets’ motion according to Kepler’s laws. Particularly, the sun and its planets (objects) moving around it in (fictitious) oval paths (orbits) are utilized to simulate the search space, which represents Kepler’s first statement. Specifically, the planets in KOA (nominated solutions) exist at various positions from the sun (optimal solution) and at different times, hence, the exploration and exploitation concepts are effectively performed, as shown in Fig. 2. There are many factors affecting the planet’s position from the optimal solution (the sun) such as the actual planet’s position, the attraction force between the sun and the planet, and its revolving speed around the sun^39^.

Principally, KOA starts with stochastic initialization of the objects’ numbers and positions, as represented by (15).

15 $${Y}_{i}^{j}={lb}_{i}^{j}+{s}_{1}\times \left({hb}_{i}^{j}-{lb}_{i}^{j}\right), \left\{\begin{array}{c}i=\mathrm{1,2},\dots ,{N}_{p}\\ j=\mathrm{1,2},\dots , d\end{array}\right.$$

where ${Y}_{i}^{j}$ denotes the ${i}^{th}$ object (nominated solution) in the search area. The population of the solution nominees in the search area is represented by ${N}_{p}$ and the number of variables to be optimized is defined by $d$. ${lb}_{i}^{j}$ and ${hb}_{i}^{j}$ are the lower and higher boundaries of the ${j}^{th}$ decision parameter, respectively.

The initialization of the orbital deviation ${O}_{d}$ for each ${i}^{th}$ object is given by (16).

16 $${{O}_{d}}_{i}={s}_{2}, i=\mathrm{1,2},\dots ,{N}_{p}$$

Lastly, the orbital interval ${O}_{t}$ for each *i*th object is initialized by (17).

17 $${{O}_{t}}_{i}=\left|s\right|, i=\mathrm{1,2},\dots ,{N}_{p}$$

where $s$ is a stochastically normal generated number.

The gravitational force ${{G}_{f}}_{i}$ that attracts any plant ${Y}_{i}$ to the sun ${Y}_{s}$ is defined by the universal gravitational law which is function of the sun and the planet mass ${m}_{s}$ and ${m}_{i}$, respectively, and length between the star and the planet ${R}_{i}$, as given by (18).

18 $${{G}_{f}}_{i}\left(t\right)={{O}_{d}}_{i}\times \alpha \left(t\right)\times \frac{\overline{{m }_{s}}\times \overline{{m }_{i}}}{{\overline{{R }_{i}}}^{2}+\epsilon }+{s}_{3}$$

where the global gravitational constant is symbolized by $\alpha$ and formulated by (19), and $\epsilon$ is a trivial number to avoid dividing by zero error. $\overline{{R }_{i}}$ defines the normalized value of ${R}_{i}$ which is calculated by (20). The normalized values of ${m}_{s}$ and ${m}_{i}$ are represented by $\overline{{m }_{s}}$ and $\overline{{m }_{i}}$ and computed by (21)–(24), respectively.

19 $$\alpha \left(t\right)={\alpha }_{o}{.e}^{\frac{-\gamma .t}{{t}_{m}}}$$

where, $t$ and ${t}_{m}$ are the actual iteration and the highest number of iterations. The initial value and a constant are denoted by ${\alpha }_{o}$ and $\gamma$, respectively.

20 $${R}_{i}\left(t\right)={\left[\sum_{j=1}^{d}{({Y}_{s}^{j}-{Y}_{i}^{j})}^{2}\right]}^{0.5}$$

21 $${m}_{s}={s}_{4}\times \frac{{bst}_{s}\left(t\right)-wst(t)}{{\sum }_{k=1}^{{N}_{p}}({f}_{k}\left(t\right)-wst(t))}$$

22 $${m}_{i}=\frac{{bst}_{i}\left(t\right)-wst(t)}{{\sum }_{k=1}^{{N}_{p}}({f}_{k}\left(t\right)-wst(t))}$$

23 $${bst}_{s}\left(t\right)=\mathrm{min}\left({f}_{k}\left(t\right)\right), k\in \mathrm{1,2},\dots ,{N}_{p}$$

24 $$wst\left(t\right)=\mathrm{max}\left({f}_{k}\left(t\right)\right), k\in \mathrm{1,2},\dots ,{N}_{p}$$

where ${s}_{4}$ is a stochastic number generated from 0 to 1. ${f}_{k}\left(t\right)$ refers to the fitness function of the *k*th object.

According to Kepler’s law, the velocity of a planet is dependent on its position from the sun. In detail, if the planet is close to the sun, it will experience a strong gravitational force, and hence, it will accelerate its motion to avoid pulling into the sun and vice versa. So, the planet’s velocity is described by (25).

25 $${v}_{i}\left(t\right)=\left\{\begin{array}{c}\vartheta .\left(2{s}_{5}.\overrightarrow{{Y}_{i}}-\overrightarrow{{Y}_{b}}\right)+\ddot{\vartheta }.\left(\overrightarrow{{Y}_{a}}-\overrightarrow{{Y}_{b}}\right)+\left(1-{{R}_{i}}_{n}\left(t\right)\right).F.\overrightarrow{{c}_{1}}.\overrightarrow{{s}_{6}}.\left(\overrightarrow{{hb}_{i}}-\overrightarrow{{hb}_{i}}\right),\\ \begin{array}{c}{{R}_{i}}_{n}\left(t\right)\le 0.5 \\ {s}_{5}.\Psi .\left(\overrightarrow{{Y}_{a}}-\overrightarrow{{Y}_{i}}\right)+\left(1-{{R}_{i}}_{n}\left(t\right)\right).F.\overrightarrow{{c}_{2}}.\overrightarrow{{s}_{6}}.\left({s}_{7}.\overrightarrow{{hb}_{i}}-\overrightarrow{{hb}_{i}}\right), elsewhere\end{array}\end{array}\right.$$

26 $$\vartheta =\overrightarrow{c}\times q\times\Psi $$

27 $$\Psi =\sqrt{\left[\alpha \left(t\right)\times ({m}_{s}-{m}_{i})\left|\frac{2}{{R}_{i}\left(t\right)+\epsilon }-\frac{1}{{a}_{i}(t)+\epsilon }\right|\right]}$$

28 $$q=({s}_{7}\times \left(1-{s}_{5}\right)+{s}_{5})$$

29 $$\overrightarrow{c}=\left\{\begin{array}{ll}0,&\quad \overrightarrow{{s}_{6}}\le \overrightarrow{{s}_{8}}\\ 1, &\quad else\end{array}\right.$$

30 $$\mathcal{F}=\left\{\begin{array}{ll}1,&\quad {s}_{5}\le 0.5\\ -1,&\quad else\end{array}\right.$$

31 $$\ddot{\vartheta }=(1-\overrightarrow{c})\times \overrightarrow{q}\times\Psi $$

32 $$\overrightarrow{q}=({s}_{7}\times \left(1-\overrightarrow{{s}_{6}}\right)+\overrightarrow{{s}_{6}})$$

33 $$\overrightarrow{{c}_{1}}=\left\{\begin{array}{ll}0, &\quad\overrightarrow{{s}_{6}}\le \overrightarrow{{s}_{5}}\\ 1,&\quad else\end{array}\right.$$

34 $$\overrightarrow{{c}_{2}}=\left\{\begin{array}{ll}0,&\quad \overrightarrow{{s}_{7}}\le \overrightarrow{{s}_{5}}\\ 1,&\quad else\end{array}\right.$$

35 $${{R}_{i}}_{n}\left(t\right)=\frac{{R}_{i}\left(t\right)-\mathrm{min}(R(t))}{\mathrm{max}\left(R\left(t\right)\right)-\mathrm{min}(R\left(t\right))}$$

where ${a}_{i}(t)$ symbolizes a semi-main axis of the oval path of the ${i}^{th}$ planet and is given by (36).

36 $${a}_{i}(t)={s}_{5}\times \sqrt[3]{\left[{{{O}_{t}}_{i}}^{2}\times \frac{\alpha \left(t\right)\times ({m}_{s}+{m}_{i})}{4{\pi }^{2}}\right]}$$

Generally, most objects move around the sun in an anti-clockwise direction, nevertheless, some of them may revolve in a clockwise motion. KOA emulates this phenomenon by employing a flag $\mathcal{F}$ to avoid trapping in the local minima zones. More specifically, KOA use $\mathcal{F}$ to adjust the search flow so that the objects enhance their scanning ability in the search space.

The exploration phase is attained when the objects are away from the sun, referring that KOA efficiently explores the whole search space. Thence, the updated position of each planet away from the sun is described by (37).

37 $$\overrightarrow{{Y}_{i}}\left(t+1\right)=\overrightarrow{{Y}_{i}}\left(t\right)+\mathcal{F}\times \overrightarrow{{v}_{i}}\left(t\right)+({{G}_{f}}_{i}\left(t\right)+\left|s\right|)\times \overrightarrow{c}\times \left(\overrightarrow{{Y}_{s}}(t)-\overrightarrow{{Y}_{i}}(t)\right)$$

On the other hand, if the objects are close to the star, KOA will concentrate on optimizing the exploitation phase. The switching between the two phases is done using an adaptive controlling factor $h$, which gradually alters as a function of the time, as revealed in (38). Thus, the new position of the planets based on this controlling strategy is formulated by (39).

38 $$h={\left[\mathrm{exp}(s(({a}_{2}-1)\times {s}_{5}+1))\right]}^{-1}$$

39 $$ \begin{gathered} \overrightarrow {{Y_{i} }} \left( {t + 1} \right) = \overrightarrow {{Y_{i} }} \left( t \right) \times \vec{c} + \left( {1 - \vec{c}} \right) \hfill \\ \times \left[ {\frac{{\overrightarrow {{Y_{i} }} \left( t \right) + \overrightarrow {{Y_{s} }} \left( t \right) + \overrightarrow {{Y_{a} }} \left( t \right)}}{3} + h \times \left( {\frac{{\overrightarrow {{Y_{i} }} \left( t \right) + \overrightarrow {{Y_{s} }} \left( t \right) + \overrightarrow {{Y_{a} }} \left( t \right)}}{3} - \overrightarrow {{Y_{b} }} \left( t \right)} \right)} \right] \hfill \\ \end{gathered} $$

where ${a}_{2}$ is a periodical regulating factor gradually decays from -1 to -2 for $\mathcal{M}$ cycles throughout the overall optimization task, as indicated in (40).

40 $${a}_{2}=-1-\left(\frac{t\%\frac{{t}_{m}}{\mathcal{M}}}{\frac{{t}_{m}}{\mathcal{M}}}\right)$$

Finally, the optimum position of the objects and the sun is determined by (41).

41 $$\overrightarrow{{Y}_{i,new}}\left(t+1\right)=\left\{\begin{array}{ll}\overrightarrow{{Y}_{i}}\left(t+1\right),&\quad f(\overrightarrow{{Y}_{i}}\left(t+1\right))\le f(\overrightarrow{{Y}_{i}}\left(t\right))\\ \overrightarrow{{Y}_{i}}\left(t\right),&\quad elsewhere\end{array}\right.$$

where ${s}_{x} ( x=1:8$) are stochastically generated numbers from 0 to 1.

**Figure 3:**
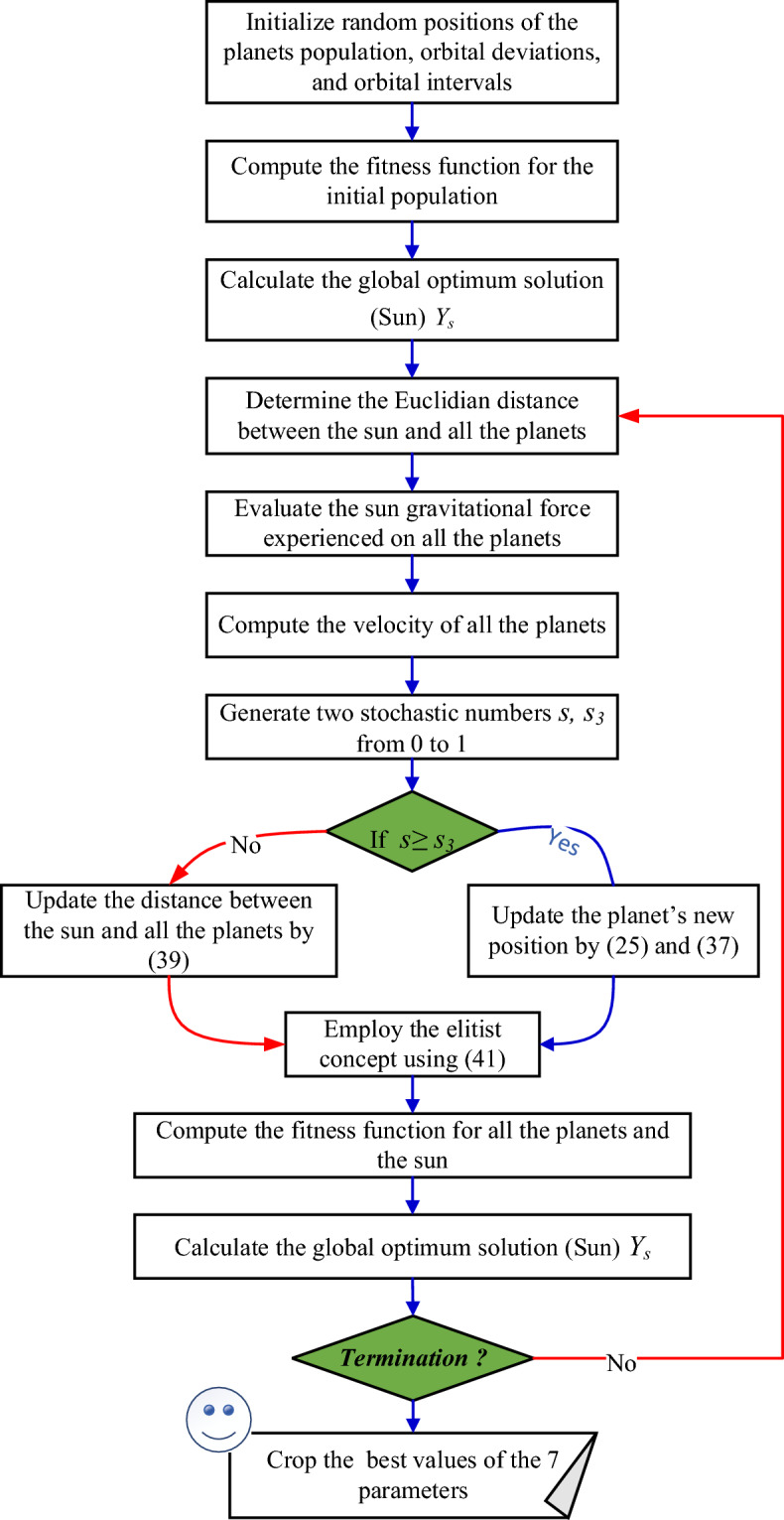
KOA’s flowchart.

It’s clearly noticed that the KOA has only to parameters need to be manually set, which are ${N}_{p}$ and ${t}_{m}$. As a result, lower computational burden and less independents runs are required to obtain the best performance of KOA. The overall steps of the KOA are presented, in detail, in Fig. 3^39^.

## Numerical simulations and algorithm verification

To appraise the robustness and the efficacy of the proposed KOA, the performance of four commercial stacks, commonly studied in the state-of-art, are assessed under different steady-state circumstances. In addition, the unknown parameters' limits have been extracted from the literature and maintained unchanged over all the test cases, to guarantee fair judgment to all the competitive algorithms. Moreover, all of the numerical simulations are carried out on a computer with an Intel Core i7 CPU and 8 GB of RAM using the MATLAB platform, version R2022a. The operating system is Windows 10 Enterprise.

It's worth asserting that the values of the KOA controlling parameters ${N}_{p}$ and ${t}_{m}$ are 10 and 10,000, respectively. Furthermore, the best values of unspecified parameters are picked after executing KOA over 20 independent runs due to the high randomness of MOAs. At the end, the statistical performance of KOA is checked to prove its robustness.

### Test cases’ datasheets and the parameters’ boundaries

The reader is invited to browse Table 1 for the technical specifications of the four earlier-announced test cases, which are extracted from^25,27–30^. The relative humidity’s of the vapor at anode and cathode ${RH}_{a}$ and ${RH}_{c}$ are maintained at 1.00 in all cases. Also, Table 1 (last three columns) reveals the minimum and maximum operating boundaries of the undefined parameters, as obtained from^34,36,37,42^.

**Table 1:** Technical specs of the test cases and practical limits of the undefined parameters.

PMEFCs’ type	Manufacture’s datasheets				Typical limits		
	Ballard Mark 5 kW	BCS 0.5 kW	NedStack 6 kW	Temasek 1 kW	Parameter	Low	High
$N$	35	32	65	20	${\zeta }_{1} (\mathrm{V})$	− 1.1997	− 0.8532
$l (\mathrm{\mu m})$	178	178	178	51	${\zeta }_{2}.{10}^{-3} (\mathrm{V}/\mathrm{K})$	1	5
${A}_{m} ({\mathrm{cm}}^{2})$	50.6	64	240	150	${\zeta }_{3}.{10}^{-5} (\mathrm{V}/\mathrm{K})$	3.6	9.8
${J}_{m} (\mathrm{A}/{\mathrm{cm}}^{2})$	1.500	0.469	1.125	1.500	${\zeta }_{4}.{10}^{-5} (\mathrm{V}/\mathrm{K})$	− 26.0	− 9.5
$T (\mathrm{K})$	343	333	343	323	$\lambda$	13	23
${P}_{H} (\mathrm{atm})$	1.0	1.0	1.0	0.5	${R}_{l} (\mathrm{m\Omega })$	0.1	0.8
${P}_{O} (\mathrm{atm})$	1.0000	0.2095	1.0000	0.5000	$\delta (\mathrm{V})$	0.0136	0.5000

### KOA-based parameters’ estimation outcomes

**Figure 4:**
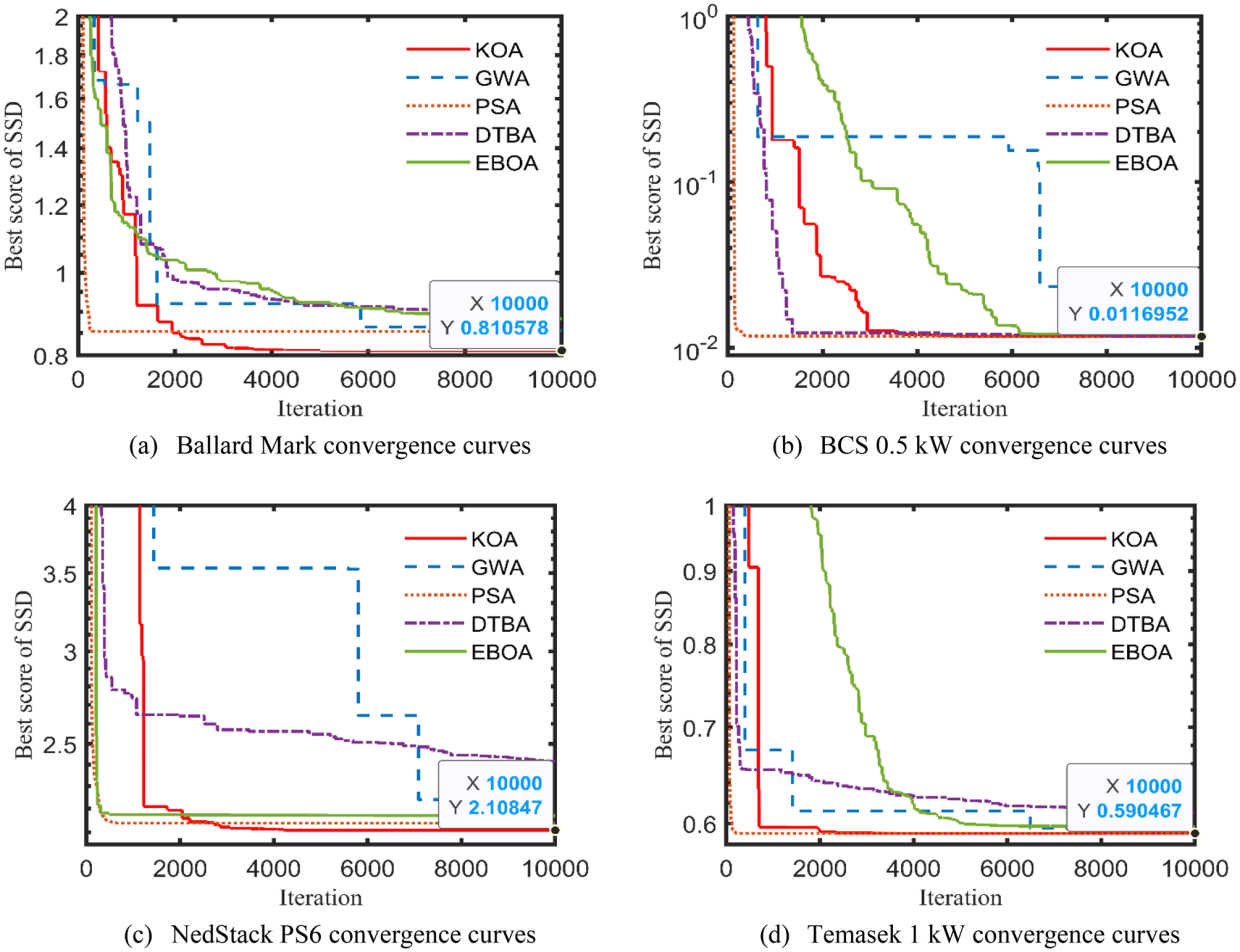
SSD convergence patterns for the test cases.

At this moment, KOA besides, two recent and two well-matured optimizers, are employed to efficiently determine the seven ungiven parameters of Amphlett’s model. Actually, the driving training-based algorithm (DTBA)^43^ and the election-based optimization algorithm (EBOA)^44^ are the two new algorithms, while grey wolf algorithm (GWA) and particle swarm algorithm (PSA) represent the two benchmark algorithms. Practically, Tables 2, 3, 4 and 5 elucidate the KOA-based minimum SSD’s values, for the four aforementioned test cases after 20 autonomous runs, compared to the four executed algorithms and the other recently-reported optimizers. Examples are improved artificial hummingbird algorithm (IAHA)^45^, honey badger algorithm (HBA)^46^, manta rays foraging algorithm (MRFA)^47^, pathfinder algorithm (PFA)^48^, neural network algorithm (NNA)^49^, and moth-flame algorithm (MFA)^50^. Besides, sparrow search algorithm (SSA)^51^, vortex search algorithm (VSA)^52^, modified monarch butterfly algorithm (MMBA)^53^, quasi oppositional bonobo algorithm (QOBA)^54^, modified farmland fertility algorithm (MFFA)^55^, marine predator algorithm (MPA)^56^, modified AEA (MAEA)^57^, satin bowerbird algorithm (SBA)^58^, and shark smell optimizer (SSO)^59^ are also brought to comparison with the proposed KOA-based results. Over and above, the optimal values of the unknown parameters for each algorithm are also captured in the above-stated tables. Furthermore, the convergence trends of the applied optimizers for the four PEMFCs’ stacks are revealed in Fig. 4a–d.

**Table 2:** KOA outcomes compared to other competitive optimizers for Ballard Mark V.

Algorithms	Parameters							
	${\zeta }_{1} (\mathrm{V})$	${\zeta }_{2}{\cdot 10}^{-3} (\mathrm{V}/\mathrm{K})$	${\zeta }_{3}\cdot {10}^{-5} (\mathrm{V}/\mathrm{K})$	${\zeta }_{4}\cdot {10}^{-5} (\mathrm{V}/\mathrm{K})$	$\lambda$	${R}_{l} (\mathrm{m\Omega })$	$\delta (\mathrm{V})$	$SSD ({\mathrm{V}}^{2})$
KOA	**− 0.8814**	**3.1030**	**7.0050**	**− 16.3984**	**23.0000**	**0.1000**	**0.0136**	**0.810578**
GWA	− 1.0102	3.2456	5.3612	− 16.3075	23.0000	0.1047	0.0136	0.855938
PSA	− 1.0635	3.3482	4.9959	− 16.2830	22.9999	0.1000	0.0136	0.853608
DTBA	− 0.8952	2.9699	5.8812	− 15.6956	23.0000	0.0175	0.0136	0.889342
EBOA	− 1.1897	3.6653	4.7175	− 15.7171	23.0000	0.0141	0.0146	0.883334
ARA^25^	− 1.1589	3.5208	4.0526	− 16.7251	23.9900*	0.1000	0.0159	0.813912
IAHA^45^	− 1.0130	4.0000	8.9800	− 16.3000	23.0000	0.1000	0.0136	0.853608
ERWCA^22^	− 0.8548	3.3043	8.8427	− 16.7251	24.0000*	0.1000	0.0159	0.813912
HBA^46^	− 1.1997	4.3345	9.2069	− 16.2830	23.0000	0.1000	0.0136	0.853610
ETSA^30^	− 0.8534	2.5591	3.6100	− 16.2868	23.0000	0.1000	0.0136	0.85360
CSA^29^	− 1.18134	3.5691	3.9929	− 16.2830	23.0000	0.1000	0.0136	0.853608
ICSO^34^	− 0.9600	Cpt	4.2500	− 17.3000	23.0000	0.1000	0.0140	0.853000
ABCDEA^23^	− 1.1956	4.2189	8.3404	− 16.2830	23.0000	0.1000	0.0136	0.853608
LSA^37^	− 1.0624	3.5970	6.6538	− 16.4925	23.0000	0.1030	0.0188	0.814000
MRFA^47^	− 1.0898	3.8249	7.7306	− 16.2830	23.0000	0.1000	0.0136	0.853300
PFA^48^	− 1.1997	3.9579	6.3901	− 16.2830	23.0000	0.1000	0.0136	0.853610
HGSA^33^	− 0.9910	3.7000	9.1000	− 16.3500	22.8700	0.1000	0.0135	0.853600
NNA^49^	− 0.9800	3.6946	9.0871	− 16.2820	23.0000	0.1000	0.0136	0.853610

*Cpt* computed.

Significant values are in bold.

*The published values violate the earlier-mentioned boundaries (see Table 1). So, an unapplicable solution.

**Table 3:** KOA outcomes compared to other competitive optimizers for BCS 0.5 kW.

Algorithms	Parameters							
	${\zeta }_{1} (\mathrm{V})$	${\zeta }_{2}{\cdot 10}^{-3} (\mathrm{V}/\mathrm{K})$	${\zeta }_{3}\cdot {10}^{-5} (\mathrm{V}/\mathrm{K})$	${\zeta }_{4}\cdot {10}^{-5} (\mathrm{V}/\mathrm{K})$	$\lambda$	${R}_{l} (\mathrm{m\Omega })$	$\delta (\mathrm{V})$	$SSD ({\mathrm{V}}^{2})$
KOA	**− 1.1654**	**3.9388**	**8.9029**	**− 19.2956**	**20.9077**	**0.1000**	**0.0161**	**0.0116952**
GWA	− 0.8534	2.5074	5.7116	− 19.3734	21.9914	0.1293	0.0166	0.0121445
PSA	− 0.9006	2.5922	5.3451	− 19.2701	21.0882	0.0136	0.0161	0.0117504
DTBA	− 0.8581	2.3739	4.7597	− 19.2863	21.9631	0.0203	0.0162	0.0117808
EBOA	− 0.9966	2.8534	5.1700	− 19.3518	22.8929	0.0216	0.0166	0.0120948
ARA^25^	− 1.1762	3.7344	7.3729	− 19.3017	20.8772	0.1000	0.0161	0.0116978
IAHA^45^	− 0.8774	3.500	9.5600	− 19.3000	20.8772	0.1000	0.0161	0.0116980
ERWCA^22^	− 1.1742	3.1597	3.7063	− 19.3017	20.8772	0.1000	0.0161	0.0116978
CSA^29^	− 1.1766	3.4965	5.8319	− 19.2897	21.3242	0.1464	0.0161	0.0117362
ABCDEA^23^	− 1.1706	4.0932	9.7961	− 19.3017	20.8772	0.1000	0.0161	0.0116978
BSA^26^	− 0.9063	2.5100	5.5000	− 15.2000	24.0000	0.1000	0.0136	4.1957000
LSA^37^	− 1.0134	2.9662	5.5693	− 19.2904	20.9300	0.1050	0.0161	0.0116900
JSA^36^	− 0.9689	2.6930	4.6700	− 19.0000	20.8389	0.1000	0.0161	0.0116990
CMSA^28^	− 0.7850	4.5000	8.8600	− 19.3000	23.0000	0.3210	0.0170	0.0120000
MFA^50^	− 1.0773	2.5000	1.4356*	− 19.2670	4.0000*	1.7447*	0.0171	0.0118110
AEA^24^	− 0.8794	2.3360	4.1100	− 19.0000	20.8755	0.1000	0.0161	0.0117000
FFA^31^	− 0.9928	2.6210	3.7464	− 19.3000	21.1011	0.1000	0.0163	0.0118190
GTA^32^	− 0.9082	3.2000	7.8620	− 19.1000	22.9584	0.4245	0.0155	0.0117000

Significant values are in bold.

**Table 4:** KOA outcomes compared to other competitive optimizers for NedStack PS6.

Algorithms	Parameters							
	${\zeta }_{1} (\mathrm{V})$	${\zeta }_{2}\cdot {10}^{-3} (\mathrm{V}/\mathrm{K})$	${\zeta }_{3}\cdot {10}^{-5} (\mathrm{V}/\mathrm{K})$	${\zeta }_{4}\cdot {10}^{-5} (\mathrm{V}/\mathrm{K})$	$\lambda$	${R}_{l} (\mathrm{m\Omega })$	$\delta (\mathrm{V})$	$SSD ({\mathrm{V}}^{2})$
KOA	**− 1.0259**	**3.3982**	**7.1661**	**− 9.5000**	**13.4430**	**0.1000**	**0.0136**	**2.10847**
GWA	− 1.0982	3.2438	4.5509	− 9.5008	13.5258	0. 1060	0.0140	2.12085
PSA	− 1.0826	3.5902	7.3410	− 9.5003	13.6172	0.0116	0.0139	2.13859
DTBA	− 0.9525	3.0469	6.1806	− 9.5000	17.5028	0.0184	0.0373	2.41624
EBOA	− 0.8532	2.3989	3.6177	− 9.5000	14.0995	0.01226	0.01795	2.16936
ARA^25^	− 1.0085	3.0434	4.9796	− 9.5400	13.4457	0.1000	0.0136	2.11125
IAHA^45^	− 0.8831	2.6000	3.6000	− 9.5000	13.4650	0.0100	0.0136	2.14570
ICSO^34^	− 0.8500	Cpt	9.7800	− 9.5600	13.3300	0.1000	0.0130	2.13900
SSA^51^	− 0.9894	3.3286	7.4100	− 9.5400	20.5477	0.2560	0.4260	2.57110
MFA^50^	− 0.8532	3.1364	8.8900	− 9.5400	13.4656	0.1000	0.0136	2.14590
MVA^24^	− 1.0394	3.2439	5.7700	− 9.5400	16.1317	0.1710	0.0290	2.36320
ALA^24^	− 0.9836	2.7815	3.6200	− 9.5400	13.9723	0.1370	0.0141	2.20340
AEA^24^	− 1.1993	4.2726	9.8000	− 9.5400	15.0028	0.1170	0.0273	2.30690
VSA^52^	− 0.8946	3.3480	9.7500	− 9.5400	13.0000	0.1030	0.0429	2.34260
MRFA^47^	− 0.9381	3.4861	9.5120	− 9.5436	13.0960	0.1000	0.0145	2.13600
MMBA^53^	− 1.0300	3.5300	8.2400	− 9.4800	15.1100	0.0164	0.0100	2.12000
FFA^31^	− 1.0357	2.9502	3.7669	− 9.5400	15.0297	0.1622	0.0136	2.16710
IFSA^42^	− 0.9200	3.4600	7.5900	− 9.6200	13.1500	0.1000	0.0400	2.15000

Significant values are in bold.

**Table 5:** KOA outcomes compared to other competitive optimizers for Temasek 1 kW.

Algorithms	Parameters							
	${\zeta }_{1} (\mathrm{V})$	${\zeta }_{2}\cdot {10}^{-3} (\mathrm{V}/\mathrm{K})$	${\zeta }_{3}\cdot {10}^{-5} (\mathrm{V}/\mathrm{K})$	${\zeta }_{4}\cdot {10}^{-5} (\mathrm{V}/\mathrm{K})$	$\lambda$	${R}_{l} (\mathrm{m\Omega })$	$\delta (\mathrm{V})$	$SSD ({\mathrm{V}}^{2})$
KOA	**− 0.8731**	**2.7642**	**6.1346**	**− 9.5000**	**13.0000**	**0.1000**	**0.1619**	**0.590467**
GWA	− 1.1996	3.9605	7.4015	− 9.5000	14.5255	0.0103	0.1729	0.594670
PSA	− 1.0615	3.5641	7.6177	− 9.5000	13.0000	0.0100	0.1619	0.590471
DTBA	− 0.8873	2.9047	6.7887	− 9.5000	20.6002	0.0175	0.1828	0.610125
EBOA	− 0.9197	2.5498	3.6751	− 9.5000	13.0000	0.0134	0.1557	0.596999
QOBA^54^	− 1.1997	3.8220	3.6000	− 22.9500	13.0000	0.1000	0.0680	0.783040
MFFA^55^	− 0.9035	3.8267	8.4751	− 22.9347	13.3251	0.1001	0.0705	0.791000
MPA^56^	− 0.9777	3.4240	4.9692	− 23.6873	10.0000	0.1000	0.0225	0.755900
CHHA^27^	− 1.0944	4.4280	8.7600	− 21.4650	18.6392	0.1891	0.1016	0.802340
MAEA^57^	− 0.8544	3.5766	7.8888	− 22.9258	13.0017	0.1000	0.0683	0.790960
SBA^58^	− 1.0312	2.4095	3.9500	− 9.5368	9.9852	0.1124	0.1269	1.632200
SSO^59^	− 1.0299	2.4105	4.0000	− 9.5400	10.0005	0.1087	0.1274	1.648100

Significant values are in bold.

The observation we may mention about KOA achieving excellent results for the studied applications but being hampered by a slight delay in convergence (required high iteration) is indeed an important limitation compared to those published in the literature. Convergence refers to the point at which an algorithm’s performance stabilizes, indicating that it has learned the underlying patterns in the data.

A delay in convergence can impact the efficiency and effectiveness of an algorithm, particularly in real-time or time-sensitive applications where prompt decision-making is crucial. However, it’s worth noting that convergence speed can vary depending on the complexity of the problem, the size of the dataset, and the specific algorithm being used.

**Figure 5:**
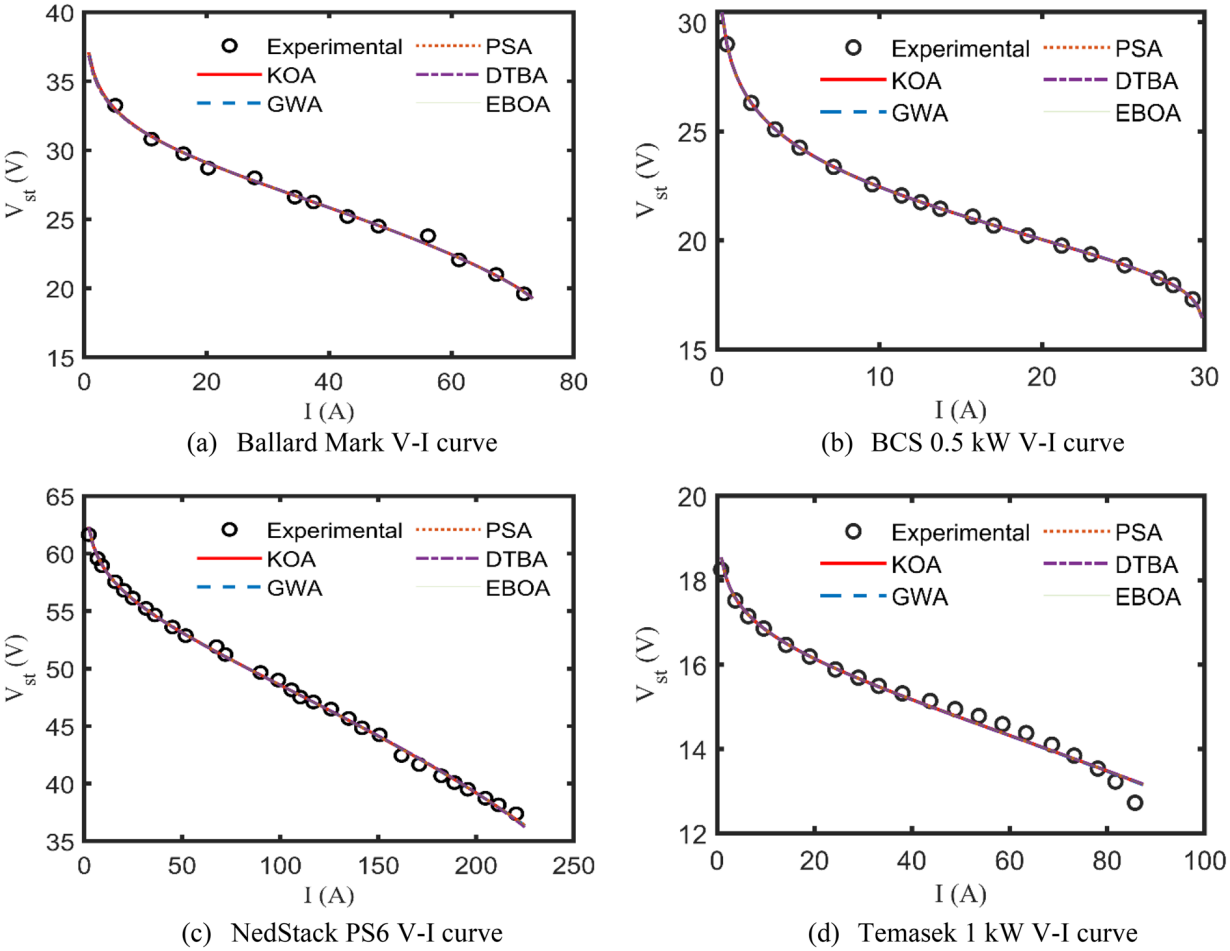
V–I curves for the four test cases.

It may be affirmed from the convergence patterns that KOA outperforms the other candidates in terms of escaping from getting trapped in local minima while maintaining the fastest rate to reach the best SSD throughout 10,000 iterations. Accordingly, the polarization (V–I) curves of the actually recorded and the KOA, GWA, PSA, DTBA, and EBOA-based simulated datasets for the four commercial PEMFCs’ stacks are captured in Fig. 5a–d. A closer look at Fig. 5a–d, it can be caught that the computed V–I curves, generated after injecting the optimal values of the parameters to the model, are consistent and well-fitted to the relevant experimental values. Furthermore, the minor SSD’s values support the afore-said statements (see Tables 2, 3, 4, 5).

**Figure 6:**
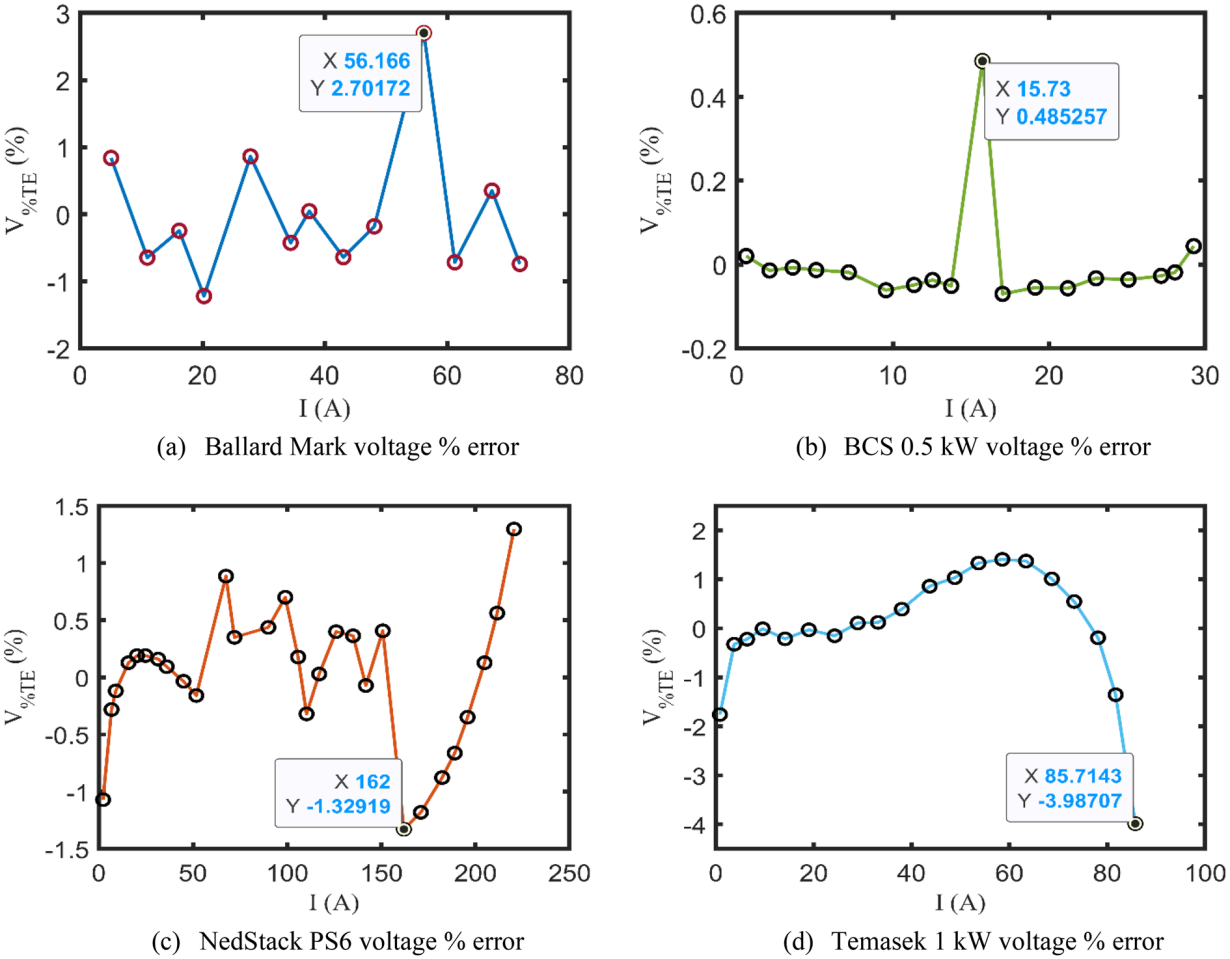
Percentage voltage error curves.

Another form of KOA verification is the percentage terminal voltage error ${V}_{\%TE}$, which is determined to appraise how reliable and accurate the KOA is to fit the model-based calculated terminal voltages to the experimental ones, as described by (42)^36,46^. Figure 6a–d elucidates the changeability of ${V}_{\%TE}$ along with the relevant stack drawn current for the four benchmark test cases. It’s worth spotlighting that the highest values of ${V}_{\%TD}$ for Ballard Mark, BCS 0.5 kW, NedStack PS6, and Temasek 1 kW are 2.7017%, 0.4853%, − 1.3292%, − 3.9871%, respectively.

42 $${V}_{\%TE}=\frac{{V}_{ms}-{V}_{cal}}{{V}_{ms}}\times 100$$

**Figure 7:**
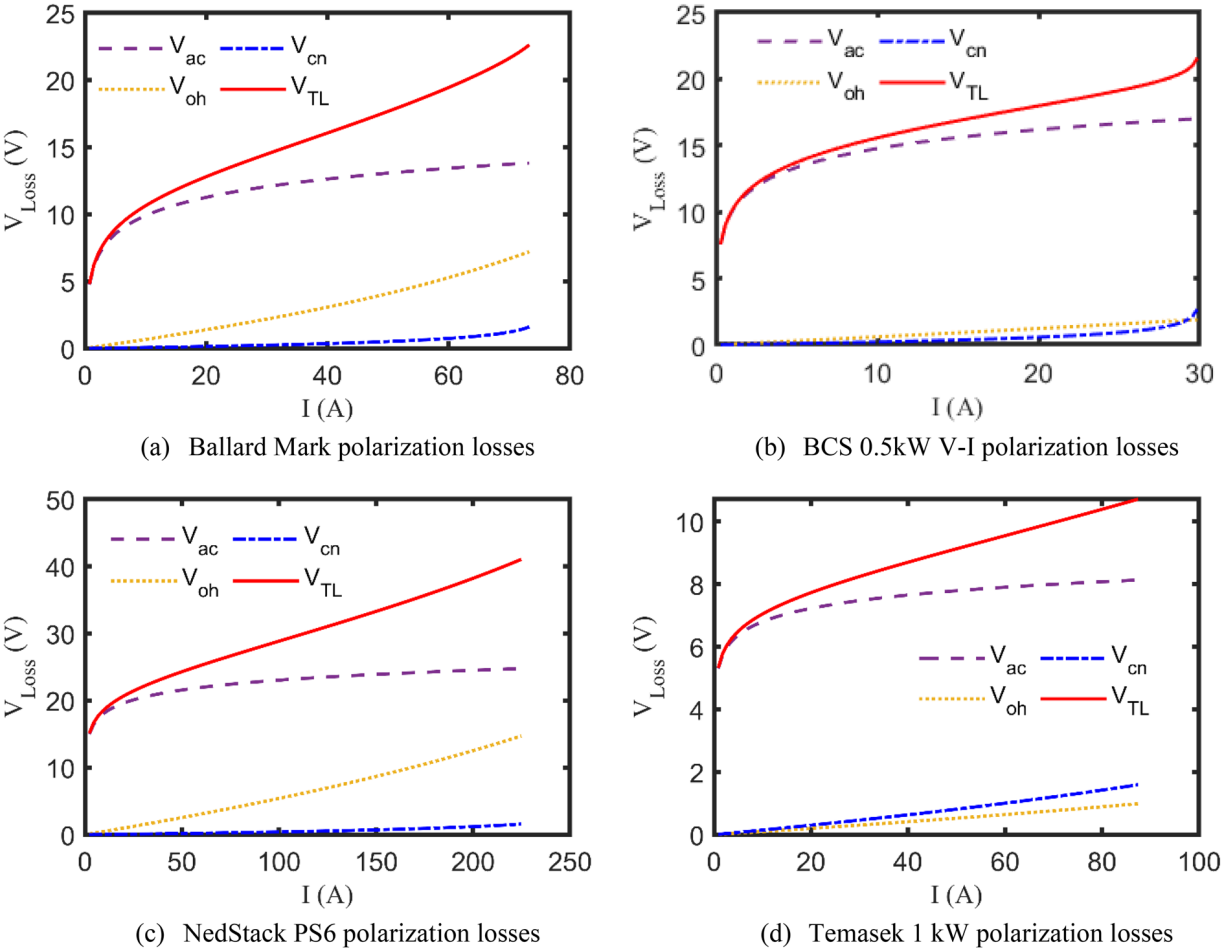
Plots of polarization losses for the test cases.

Now, after validating the proposed KOA-based methodology, it’s time to utilize its outcomes to study the impact of the polarization losses on the PEMFCs’ voltage profile. As previously discussed, the terminal voltage of the PEMFCs’ stack is influenced by three voltage drops; activation, ohmic, and concentration losses^54,57^. In this regard, Fig. 7a–d reveal the alternation of every single polarization loss besides, the total losses ${V}_{TL}$ as functions of the stack drawn current, for all test cases. It can be concluded from Fig. 7a–d that when starting the PEMFCs at light load, the activation losses rapidly increase, then at intermediate loading values, it almost gets saturated, while the ohmic losses start a linear rise. At heavy drawn currents, the concentration losses considerably arise.

### KOA-based outcomes under various operating conditions

**Figure 8:**
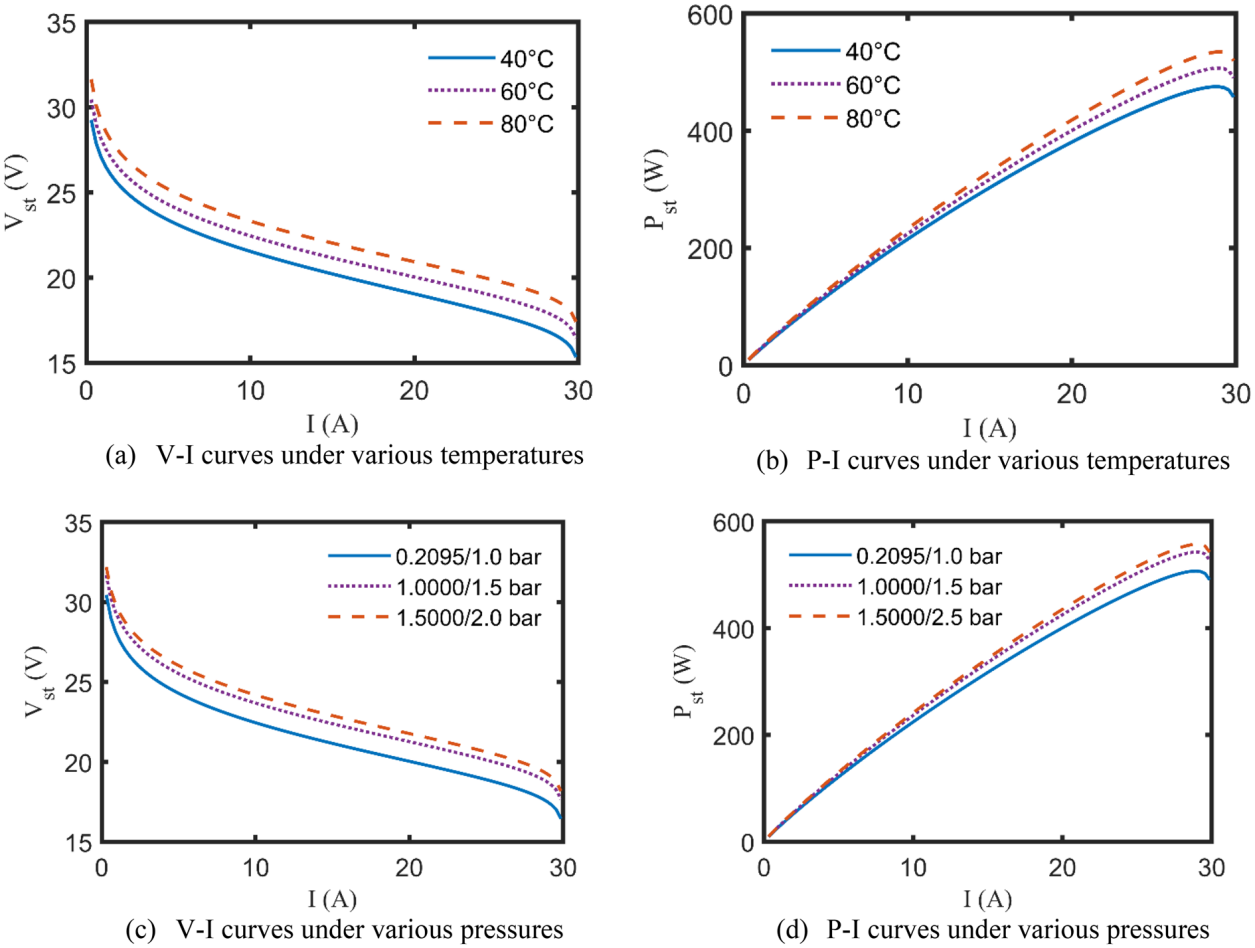
V–I and P–I curves of BCS 0.5 kW under various operating conditions.

**Figure 9:**
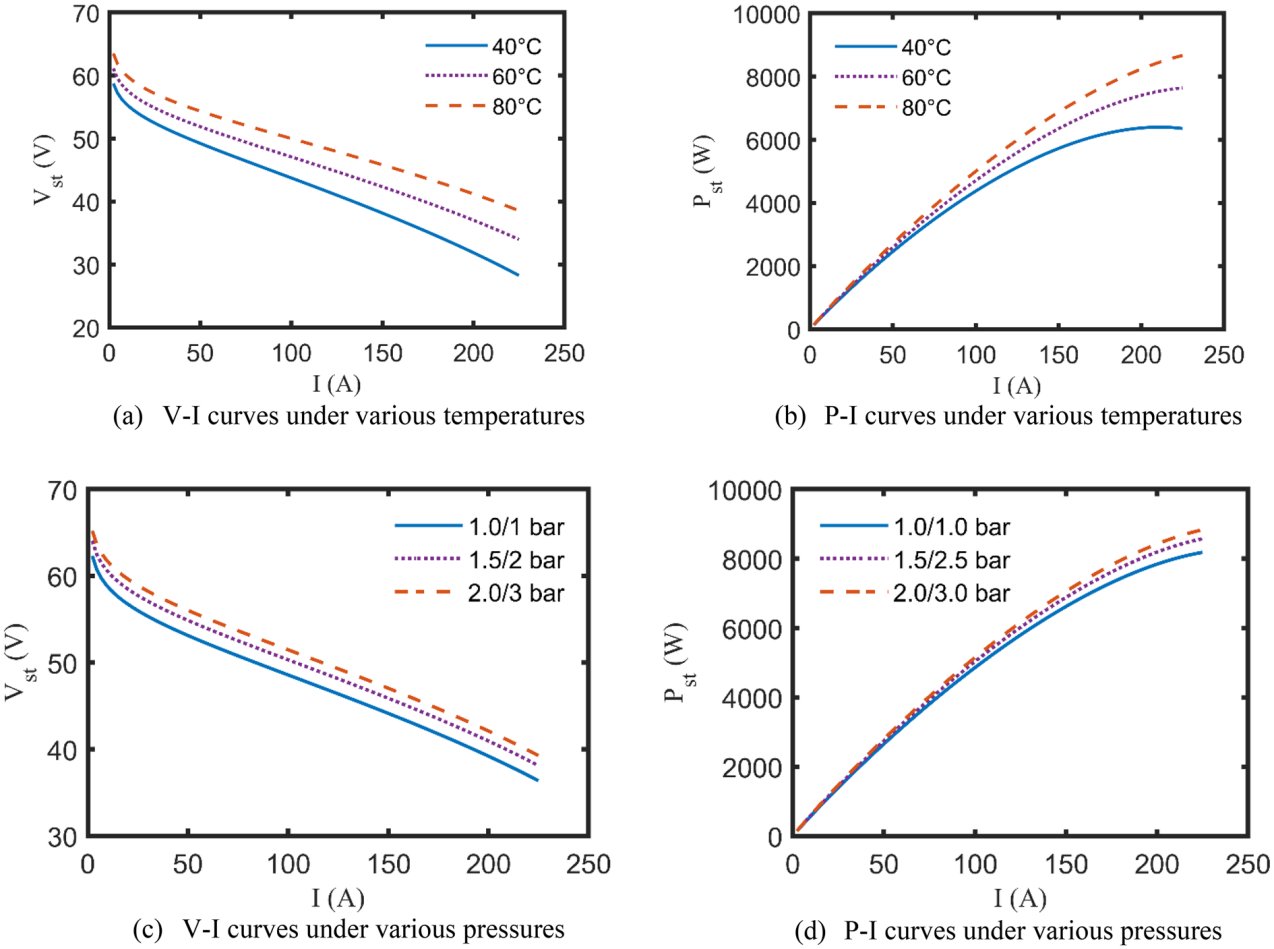
V–I and P–I curves of NedStack 6 kW under various operating conditions.

In this subsection, after accrediting the KOA-based results, the effect of varying the PEMFCs’ operating factors, ${P}_{O}/{P}_{H}$ and $T$, on the polarization characteristics (V–I and P–I curves) can be adequately evaluated. Particularly, only two test cases are addressed for this purpose to avoid a lengthy article. More specifically, The P–I and V–I curves of BCS 0.5 kW and NedStack 6 kW under changing the temperature (40, 60, and 80 °C), while maintaining the other factors constant, are caught in Figs. 8a,b and 9a,b, respectively. Moreover, Fig. 8c,d illustrates the impact of adjusting the suppliant partial pressures (${P}_{O}/{P}_{H}$ = 0.2095/1, 1/1.5, 1.5/2.5 bar) on the aforementioned curves of BCS 0.5 kW, while unchanging the other datasheet’ factors. The same is done for NedStack 6 kW, the V–I and P–I curves are generated while varying the input partial pressures (${P}_{O}/{P}_{H}$ = 1/1, 1.5/2, 2/3 bar), as revealed in Fig. 9c,d^25,36^.

It goes without saying that according to Figs. 8 and 9, increasing the operating factors of the PEMFCs, whether ${P}_{O}/{P}_{H}$ or $T$, positively affects the stack output voltage and power at the same drawn current. However, any increase over the nominal limits stated by the manufacturer will lead to severe degeneration of PEMFC’s performance.

### KOA’s statistical performance evaluation

Here, several statistical indices are generated to examine the computational performance of the proposed KOA. Mainly, the min, the max, the mean, and the standard deviation (StD) of the SSD are calculated after 20 independent runs for all the implemented algorithms. Hence, Table 6 depicts the values of those statistical parameters of KOA, GWA, PSA, DTBA, and EBOA for the four illustrated test cases. Since the parameters’ estimation of PEMFC is an offline task, which means that the parameters shall be determined before operating the PEMFC, it’s insignificant to consider the algorithms’ computational time. Nevertheless, according to Table 6 (last column), KOA extremely outperforms the other competitors in terms of the computational burden.

**Table 6:** Statistical indices of KOA and others.

Type	Algorithm	Indices				Elapsed time (s)
		Min	Mean	Max	StD	
Ballard Mark	KOA	**0.810578**	**0.810579**	**0.810588**	**3.155338e−06**	**16.672351**
	GWA	0.855938	0.895411	1.004077	4.390642e−02	86.378611
	PSA	0.853608	0.930678	1.542673	2.165230e−01	176.996924
	DTBA	0.889342	0.910242	0.939791	1.445920e−02	243.268816
	EBOA	0.883334	0.914861	0.971580	2.927553e−02	66.193528
BCS 0.5 kW	KOA	**0.011695**	**0.011727**	**0.011796**	**3.645488e−05**	**23.667014**
	GWA	0.012144	0.015062	0.021112	2.734507e−03	111.760233
	PSA	0.011750	0.016711	0.022699	4.304852e−03	237.138144
	DTBA	0.011781	0.012891	0.015595	1.486775e−03	338.538775
	EBOA	0.012095	0.012820	0.014914	9.297347e−04	95.803224
Nedstack PS6	KOA	**2.108470**	**2.135662**	**2.329817**	6.956982e−02	**35.459936**
	GWA	2.120850	2.341838	2.924057	2.549744e−01	179.916095
	PSA	2.138593	2.382487	2.677051	1.755165e−01	293.776877
	DTBA	2.416243	2.460612	2.495732	**3.004563e−02**	523.256794
	EBOA	2.169365	2.351896	2.682705	1.752544e−01	159.706073
Temasek 1 kW	KOA	**0.590467**	**0.590467**	**0.590467**	**2.309102e−09**	**24.871057**
	GWA	0.594670	0.655523	0.729511	5.770937e−02	126.789319
	PSA	0.590471	0.604481	0.642790	1.751757e−02	185.479748
	DTBA	0.610125	0.652513	0.731426	4.939069e−02	359.479300
	EBOA	0.596999	0.616389	0.642364	1.442578e−02	97.767673

Significant values are in bold.

Table 6 indicates that KOA outperforms the other implemented optimizers in terms of statistical performance. All the afore-announced results appraise how efficient, robust, and fast the KOA-based methodology is to tackle the parameters’ determination problem of Amphlett’s well-known steady-state model. This motivates the authors to examine these outcomes on the dynamic response of the PEMFCs, as illustrated in the next section.

In addition, a further analysis to indicate the time complexity of the implemented algorithms using Big O(…) is performed. In which, the time complexities of the KOA, GWA, PSA, DTBO and EBOA algorithms can be expressed as *O(N.T*_*max*_*)*, *O(N.T*_*max*_*.f)*, *O(N.T*_*max*_*.f)*, *O(N.m.(1* + *3T*_*max*_*))*, and *O*(*N.m.*(1 + 2*T*_*max*_)), respectively. *N* is the population size, *T*_*max*_ is the number of iterations, *m* is the number of problem variables, and *f* represents the time complexity of evaluating the objective function for a single particle. The above mentioned justified that why the elapsed time for processing of KOA is lesser than others as indicated in Table 6.

Regarding the computational cost, it’s true that some metaheuristic algorithms can be computationally expensive, the actual computational cost depends on various factors such as problem size, convergence criteria, and implementation efficiency. It is important to evaluate the trade-off between computational cost and the quality of the obtained solution as indicated above. Bear in mind that the task of PEMFC’s parameters estimation is off-line in nature.

## Dynamic assessment of PEMFCs stack

In this context, the simplified dynamic performance of the PEMFCs stack is evaluated from the electrical perspective. Since the time constant of the PEMFC chemical reactions is almost 10^–19^ s, the electrochemical response can be neglected. Besides, the polarization losses are assumed uninfluenced by the PEMFC’s load dynamics, except for the activation losses. Thence, the mathematical representation of the dynamic activation voltage drop ${V}_{ac}(t)$ is given by (43)^23,57^.

43 $${V}_{ac}(t)={V}_{ac}(see \left(7\right))\times \frac{1}{{t}_{d}.{e}^{\frac{-t}{{t}_{d}}}+1}$$

where, the time constant of the activation over-potential reaction is symbolized by ${t}_{d}$.

**Figure 10:**
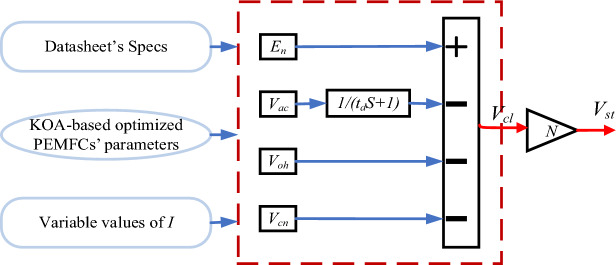
Block diagram of the simplified dynamic model.

It’s worth stating that this simplified version of Amphlett’s model doesn’t consider the time constants of the balance-of-plant devices, like the hydrogen and oxygen compressors^24,60–62^. Based on the above hypotheses, a simplified dynamic model is constructed via SIMULINK environment to simulate the PEMFC’s electrical performance due to load variations, as shown in Fig. 10.

**Figure 11:**
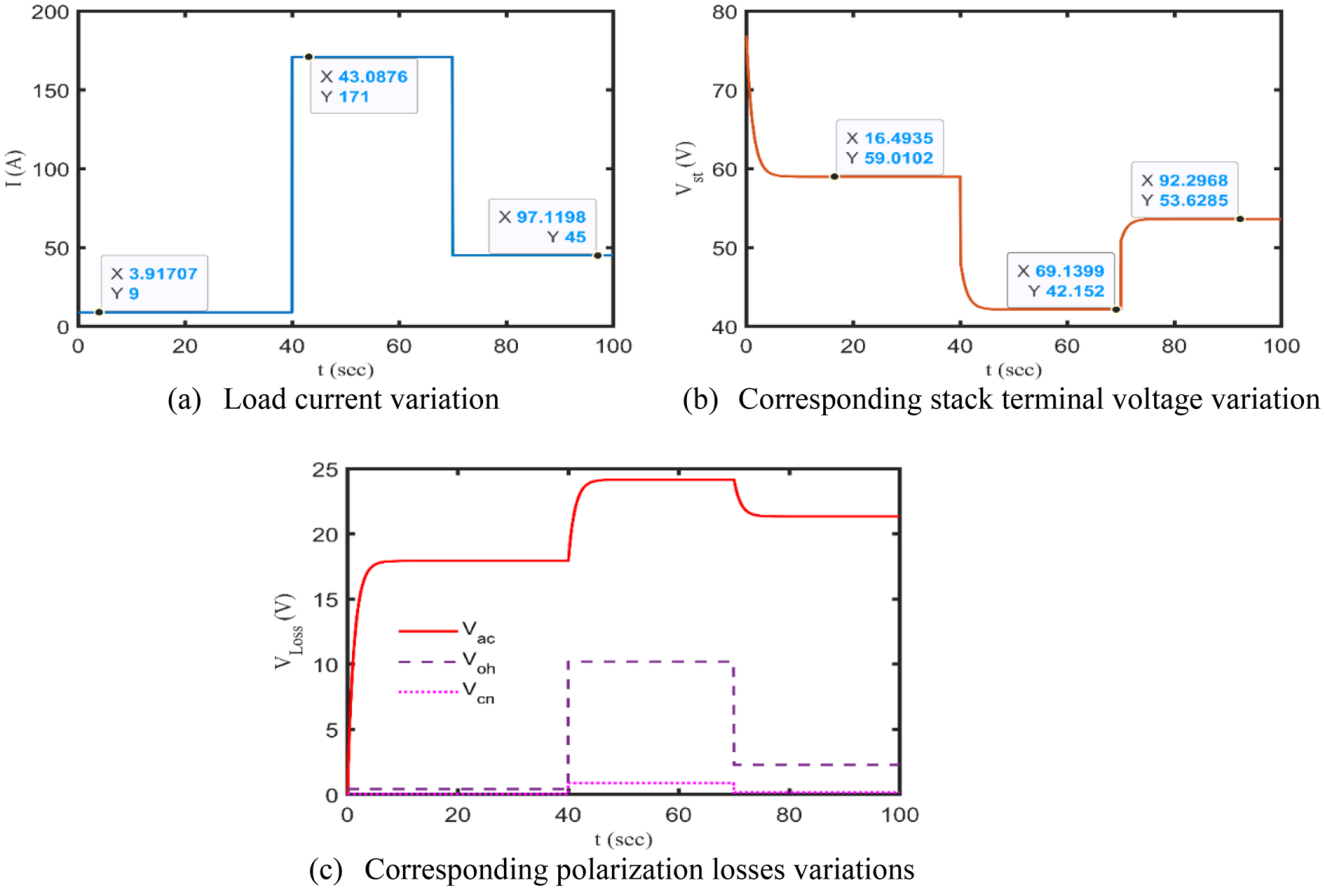
Dynamic behavior of NedStack PS6 due to sudden load variations.

As a representative of the other test cases, NedStack 6 kW stack is employed to check the validity of the proposed dynamic model, where ${t}_{d}$ equals 1.2 s and the other datasheet’s parameters are kept at their nominal values (see Table 1 (fourth column)). Specifically, a step variation of the stack drawn current, whose values are equal to 9 A, 171 A, 45 A at 0 s, 40 s, and 70 s, respectively, is applied to the proposed model, as cropped in Fig. 11a. The dynamic model simultaneously responds to this variation, as revealed in Fig. 11b,c.

More specifically, the stack terminal voltage values due to these changes are captured in Fig. 11b, which totally fit to the simulated steady-state values indicated in Fig. 5c. Furthermore, the behaviors of the internal polarization losses due to the afore variation are depicted in Fig. 11c. It’s noteworthy to recall what is stated before, as the load current increases, the stack polarization losses also increase, and accordingly its terminal voltage reduces until reaches a steady-state value as plotted in Fig. 5c. Now, it comes without any doubt that the simplified model based on the KOA’s results can effectively and acceptably simulate the electrical dynamic response of the PEMFCs.

## Conclusion and future prospective

A viable and effective methodology based on KOA has been introduced for optimally specifying the undefined parameters of the commonly investigated PEMFC’s model, called Amphlett’s model. Four practical study cases of well-known commercial PEMFCs’ types have been comprehensively discussed through a set of simulated electrical characteristics such as the calculated V–I and P–I curves and the polarization losses alternation with the drawn currents. In addition to that it’s worth mentioning that the percentage terminal voltage error between the KOA-based computed voltages and the experimentally recorded ones are 2.7%, 0.49%, − 1.33%, − 3.99% for Ballard Mark V, BCS 0.5 kW, NedStack PS6, and Temasek 1 kW PEMFCs, respectively. Furthermore, the effect of varying the input operating factors of the PEMFCs, like the temperature and the suppliant pressures, is deeply assessed for the whole test cases. Statistically, KOA robustness and preciseness have been validated through multiple indices like StD, min, max, mean, and computational CPU time, where it extremely outperforms the other challenging competitors. Moreover, the electrical dynamic performance of the PEMFCs is brought under study using an upgraded version of Amphlett’s model. This work still needs to be expanded in order to accurately analyze the performance of PEMFCs stack due to actual disturbances and other operating parameters like the hydrogen and oxygen flow rates. Additionally, the electrical behavior of the PEMFCs stacks when operating in parallel is taken into account in the anticipated future work. Once again, the promising results of the KOA encourage the research community to extend this current to study the performance of such PEMFCs stacks with maximum power tracker in real conditions and to study their behaviors' when they are connected to microgrid.

## Data Availability

The data that support the findings of this study are available from the corresponding author upon reasonable request.
